# iNOS activity is necessary for the cytotoxic and immunogenic effects of doxorubicin in human colon cancer cells

**DOI:** 10.1186/1476-4598-8-108

**Published:** 2009-11-19

**Authors:** Sara De Boo, Joanna Kopecka, Davide Brusa, Elena Gazzano, Lina Matera, Dario Ghigo, Amalia Bosia, Chiara Riganti

**Affiliations:** 1 Department of Genetics, Biology and Biochemistry, University of Turin, via Santena 5/bis, 10126 Turin, Italy; 2 Department of Internal Medicine, Laboratory of Tumour Immunology, University of Turin, corso Dogliotti 14, 10126 Turin, Italy; 3 Research Center on Experimental Medicine (Ce.R.M.S.), University of Turin, via Santena 5/bis, 10126 Turin, Italy

## Abstract

**Background:**

Doxorubicin is one of the few chemotherapeutic drugs able to exert both cytotoxic and pro-immunogenic effects against cancer cells. Following the drug administration, the intracellular protein calreticulin is translocated with an unknown mechanism onto the plasma membrane, where it triggers the phagocytosis of tumour cells by dendritic cells. Moreover doxorubicin up-regulates the inducible nitric oxide (NO) synthase (iNOS) gene in cancer cells, leading to huge amounts of NO, which in turn acts as a mediator of the drug toxicity and as a chemosensitizer agent in colon cancer. Indeed by nitrating tyrosine on the multidrug resistance related protein 3, NO decreases the doxorubicin efflux from tumour cells and enhances the drug toxicity. It is not clear if NO, beside playing a role in chemosensitivity, may also play a role in doxorubicin pro-immunogenic effects. To clarify this issue, we compared the doxorubicin-sensitive human colon cancer HT29 cells with the drug-resistant HT29-dx cells and the HT29 cells silenced for *iNOS *(HT29 *iNOS*^-^).

**Results:**

In both HT29-dx and HT29 *iNOS*^- ^cells, doxorubicin did not induce NO synthesis, had a lower intracellular accumulation and a lower toxicity. Moreover the drug failed to promote the translocation of calreticulin and the phagocytosis of HT29-dx and HT29 *iNOS*^-^cells, which resulted both chemoresistant and immunoresistant. However, if NO levels were exogenously increased by sodium nitroprusside, the chemosensitivity to doxorubicin was restored in HT29 *iNOS*^-^cells. In parallel the NO donor per se was sufficient to induce the exposure of calreticulin and to increase the phagocytosis of HT29 *iNOS*^- ^cells by DCs and their functional maturation, thus mimicking the pro-immunogenic effects exerted by doxorubicin in the parental drug-sensitive HT29 cells.

**Conclusion:**

Our data suggest that chemo- and immuno-resistance to anthracyclines are associated in colon cancer cells and rely on a common mechanism, that is the inability of doxorubicin to induce *iNOS*. Therefore NO donors might represent a promising strategy to restore both chemosensitivity and immunosensitivity to doxorubicin in resistant cells.

## Background

Despite the improvement in survival, many advanced solid cancers still remain difficult to treat. Chemotherapy, which has high success rates in certain tumours, may not succeed in removing all the cancer cells of solid tumours when these cells are affected by multidrug resistance (MDR), a multiple cross-resistance towards different anticancer drugs [1]. Several mechanisms account for the MDR phenotype, such as the reduced uptake or the increased efflux of the drug, the genetic modification of the drug's specific targets, the increased ability to repair DNA damage or to inactivate the drug through detoxification [2]. Several efflux pumps, e.g. the P-glycoprotein (Pgp) and the MDR-related proteins (MRPs), are often over-expressed on the membrane of MDR cells and mediate the efflux of a great variety of anticancer agents, such as anthracyclines, Vinca alkaloids, epipodophyllotoxins, taxanes, actinomycin-D and mitoxantrone, leading to a poor prognosis [1].

Chemotherapy usually induces the cancer cells to die in an apoptotic way, which is poorly immunogenic. However anthracyclines, in particular doxorubicin, have been found to be the only chemotherapeutic agents which elicit also an immunogenic death in tumour cells. Before inducing the apoptosis of tumour cells, doxorubicin has been shown to induce the translocation of calreticulin (CRT), a calcium sensor protein which resides in the endoplasmic reticulum (ER), to the plasma membrane [3]. After such an exposure, CRT may trigger the recognition and the phagocytosis of dying cancer cells by dendritic cells (DCs) [3,4]. CRT was found to be only exposed on cells that succumb to immunogenic cell death but was lacking on the surface of cells which undergo a non-immunogenic cell death. Therefore this protein has been considered determinant for an immuno-mediated cell death [3,4]. Interestingly, recombinant CRT has been successfully used as an anti-tumour vaccination in immunocompetent mice when injected with cancer cells treated *ex vivo *with anthracyclines [4].

Besides inducing CRT translocation to the membrane, doxorubicin was also found to be the only chemotherapeutic agent that induces the production of nitric oxide (NO) [5]. NO is a small signalling molecule synthesized by three NO synthases (NOS; EC 1.14.13.39) isoforms, two of which (neuronal NOS/nNOS or NOS I; endothelial NOS/eNOS or NOS III) are mainly constitutive. The third NOS isoform (NOS II or inducible NOS, iNOS) is virtually absent in resting cells but is up-regulated by several stimuli, like cytokines and bacterial lipopolysaccharide [6]. Doxorubicin itself is a strong *iNOS *inducer [7,8], by favouring the degradation of the IkBα inhibitory complex and allowing the NF-κB factor to translocate into the nucleus and activate the transcription of *iNOS *gene [9]. NO plays an important role in cell growth, differentiation and apoptosis [6] and it has been suggested that at least part of the doxorubicin cytotoxic effect is due to the increased NO synthesis elicited by the drug [7].

Our group reported earlier that doxorubicin induces NF-κB activation and NO synthesis in human colon cancer HT29 cells, but fails to augment the levels of NO in the drug-resistant HT29-dx sub-clone, where the drug is pumped out of the cell before it can elicit any nuclear translocation of NF-kB and increase of iNOS expression [5,9]. Moreover NO itself was found to modulate the activity of Pgp and MRP3 transporters, for which doxorubicin is a substrate [1]: indeed NO lowers the Vmax of doxorubicin efflux by nitrating the MRP3 transporter in human colon cancer HT29 cells [5,9]. Also in drug-resistant cells, if NO levels are increased by *iNOS *inducers other than doxorubicin or by NO donors, Pgp and MRP3 proteins are inhibited and the doxorubicin accumulation and toxicity are increased, resulting in a reversion of the MDR phenotype [5,9-11].

Notably, NO may also regulate the translocation of proteins from ER to the plasma membrane: for example it has been found to induce the exposure of glucose-transporter 4 onto the plasma membrane [12]. Like CRT, most glucose-transporter 4 molecules reside within the intracellular vesicular compartment until they are stimulated by insulin and NO to translocate on cell surface [12].

Since doxorubicin is the only anticancer agent which induces both NO synthesis and calreticulin translocation, we hypothesized that NO may be the agent by which doxorubicin induces the exposure of CRT, thus acting as an intracellular mediator of the doxorubicin pro-immunogenic effects. Moreover we raised the question whether chemoresistance and immunoresistance would be associated in cancer cells: since in chemo-resistant cells doxorubicin fails to induce NO synthesis and cytotoxic effects, we wondered whether the drug also fails to induce calreticulin translocation in MDR cells and whether this failure may be due to the lack of NO increase. To this purpose, we knocked down *iNOS *gene by small interfering RNA (siRNA) in human colon doxorubicin-sensitive HT29 cells and we evaluated the doxorubicin toxic and pro-immunogenic effects in drug-sensitive HT29 cells, drug-resistant HT29-dx cells and doxorubicin-sensitive HT29 cells subjected to *iNOS *silencing (termed "HT29 *iNOS*^- ^cells"). Finally we assessed whether the restoration of NO levels alone was sufficient to rescue the doxorubicin cytotoxicity and the immunogenic death in HT29 *iNOS*^- ^cells.

## Results

### Doxorubicin fails to induce NO synthesis in HT29-dx and HT29 *iNOS*^- ^cells

**Figure 1:**
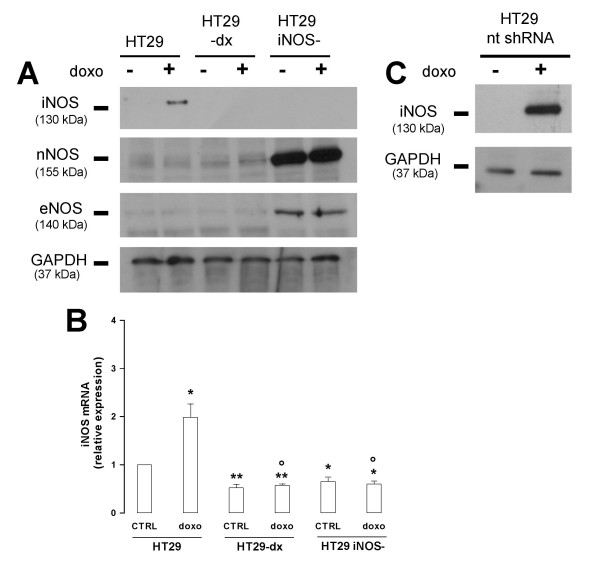
***iNOS *silencing in HT29 cells**. (**A**) Western blot detection of iNOS, eNOS, nNOS and GAPDH in HT29, HT29-dx and HT29 *iNOS*^- ^cells. The silencing of *iNOS *was performed in HT29 cells as reported under the Methods section; then, after a 24 h incubation in the absence (-) or in the presence (+) of 5 μmol/L doxorubicin, cells were lysed and Western blotting was performed as described. The expression of the housekeeping protein GAPDH was measured as equal control loading. The results shown here are representative of three similar experiments. (**B**). Real Time-PCR for *iNOS *in HT29, HT29-dx and HT29 *iNOS*^- ^cells. After a 24 h incubation in the absence (*CTRL*) or in the presence of 5 μmol/L doxorubicin (*doxo*), cells were lysed and Real Time-PCR was performed as described in the Methods section. Data are represented as mean ± SE (n = 3). Vs HT29 CTRL: * *p *< 0.01; ** *p *< 0.005. Vs HT29 doxo: ° *p *< 0.02. (**C**) HT29 cells were transfected with a non-targeting scrambled shRNA sequence (*nt shRNA*), then incubated in the absence (-) or in the presence (+) of 5 μmol/L doxorubicin for 24 h and subjected to Western blot for iNOS and GAPDH.

After silencing the *iNOS *gene by small RNA interference in HT29 cells, as reported under the Methods section, we checked the expression of the three NOS isoforms in the wild-type HT29 cells, in the drug-resistant HT29-dx cells and in the HT29 *iNOS*^- ^cells. In untreated cells, iNOS protein was absent in the three cell lines, but was greatly increased by doxorubicin in HT29 cells. On the opposite the drug failed to induce iNOS expression in HT29-dx and HT29 *iNOS*^- ^cells (Fig. 1A). Real Time-PCR experiments confirmed these results, showing that doxorubicin significantly augmented the mRNA transcript of *iNOS *only in HT29 cells (Fig. 1B). *iNOS *mRNA was even lower in control HT29-dx and HT29 *iNOS*^- ^cells compared to HT29 cells (Fig. 1B). As far as the other NOS isoforms are concerned, HT29 and HT29-dx showed an equally low basal expression of eNOS and nNOS proteins, whereas HT29 *iNOS*^- ^displayed an overexpression of both eNOS and nNOS (Fig. 1A). Doxorubicin did not change the amount of eNOS and nNOS in all the cell lines. To assess that our gene silencing procedure was devoid of any unspecific targeting, we transfected HT29 cells with a scrambled non-targeting shRNA and we measured the expression of iNOS protein in the absence and presence of doxorubicin: as shown in Figure 1C, doxorubicin was still able to induce iNOS in cells transfected with the scrambled sequence, as it did in non-silenced HT29 cells.

**Figure 2:**
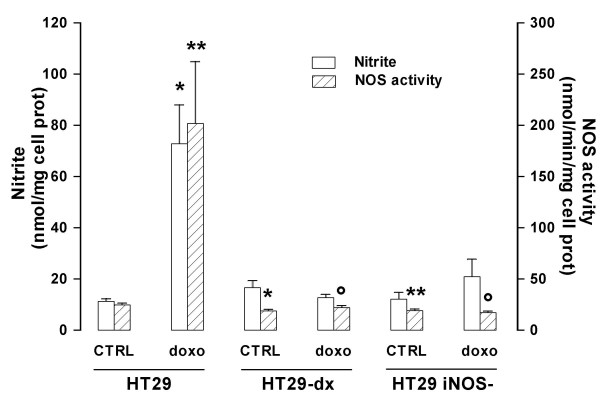
**Nitrite levels and NOS activity in HT29, HT29-dx and HT29 *iNOS*^- ^cells**. After a 24 h incubation in the absence (*CTRL*) or in the presence of 5 μmol/L doxorubicin (*doxo*), NOS activity was measured in cell lysates, and extracellular medium was checked for nitrite concentration (see Methods section). Measurements were done in duplicate and data are represented as mean ± SE (n = 4). Versus HT29 CTRL: * *p *< 0.01; ** *p *< 0.02; versus HT29 doxo: ° *p *< 0.02.

As a further confirmation of iNOS knockdown efficacy we measured the NOS activity in the cell lysate and the accumulation of nitrite, the stable derivative of NO, in the extracellular medium, taken as an index of the actual production of NO by the whole cultured cells. Both NOS activity and nitrite amount were significantly increased in HT29 cells incubated with doxorubicin, but not in HT29-dx nor in HT29 *iNOS*^- ^cells (Fig. 2). NOS activity was also lower under basal conditions in HT29-dx and HT29 *iNOS*^- ^cells compared to HT29 cells, although nitrite production showed no difference (Fig. 2).

### HT29 *iNOS*^- ^cells are as resistant to doxorubicin accumulation and toxicity as HT29-dx cells

**Figure 3:**
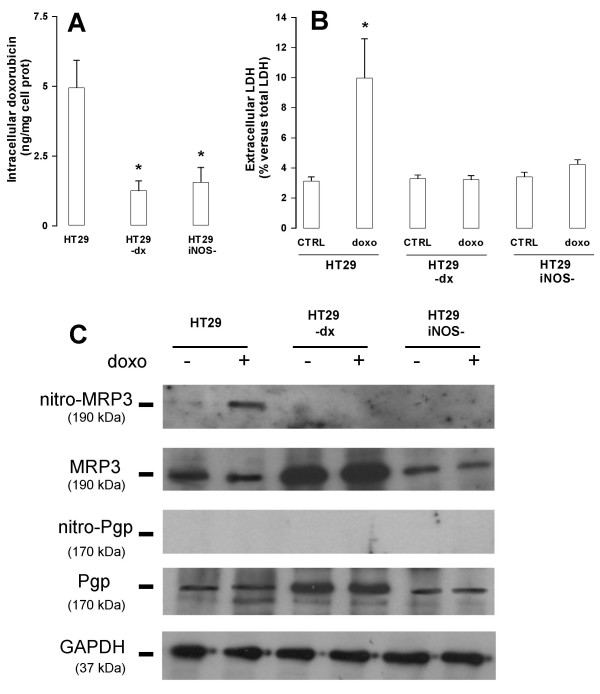
**Intracellular accumulation and cytotoxicity of doxorubicin in HT29, HT29-dx and HT29 *iNOS*^- ^cells**. (**A**) Cells were grown in RPMI containing 5 μmol/L doxorubicin for 24 h, then the drug accumulation was measured as described in the Methods section. Measurements were done in duplicate and data are represented as mean ± SE (n = 3). Versus HT29 CTRL: * *p *< 0.02. (**B**) After an incubation of 24 h in the absence (*CTRL*) or in the presence of 5 μmol/L doxorubicin (*doxo*), LDH activity was measured in the extracellular medium, as reported under the Methods section. Measurements were done in triplicate and data are represented as mean ± SE (n = 4). Versus HT29: * *p *< 0.05. (**C**) Western blot detection of nitrated MRP3, total MRP3, nitrated Pgp, total Pgp and GAPDH. HT29, HT29-dx and HT29-*iNOS*^- ^cells were incubated for 24 h in the absence (-) or in the presence (+) of 5 μmol/L doxorubicin, then cellular lysates were immunoprecipitated with an anti-nitrotyrosine polyclonal antibody. Western blotting for MRP3 and Pgp was performed on the immunoprecipitated proteins (see Methods for details). Under the same experimental conditions, an aliquot of cells was lysed and directly probed with an anti-Pgp or an anti-MRP3 antibody to detect total MRP3 or Pgp. The expression of the housekeeping protein GAPDH was measured as equal control loading. The results shown here are representative of two similar experiments.

After incubation with doxorubicin HT29-dx and HT29 *iNOS*^- ^cells showed respectively a 75% and 69% lower intracellular accumulation of the drug in comparison to HT29 cells (Fig. 3A). The extracellular LDH activity, measured as an index of the drug cytotoxicity, was significantly augmented in HT29 cells treated with doxorubicin, while it did not significantly change in HT29-dx and HT29 *iNOS*^- ^cells (Fig. 3B).

NO has previously shown to modulate the doxorubicin accumulation by nitrating the MRP3 protein [5], slowing down the efflux of the drug and making cells more sensitive to the doxorubicin cytotoxic effects. Therefore, as a possible explanation for the differences in drug accumulation and toxicity, we checked the presence of nitrated tyrosine residues on the MRP3 protein in our cell lines. Doxorubicin induced a tyrosine nitration of MRP3 in HT29 cells (Fig. 3C), the only cell line where it evoked a significant synthesis of NO (Fig. 2). On the other hand, the drug failed to promote nitration of MRP3 in both HT29-dx and HT29 *iNOS*^- ^cells (Fig 3C), where NO levels remained low (Fig 2). As far as the total level of MRP3 is concerned, the transporter was present in HT29 and HT29 *iNOS*^-^cells at similar amounts, was over-expressed in HT29-dx cells and was unaffected by the presence of doxorubicin in all the three cell lines (Fig. 3C). A similar pattern of expression was observed for Pgp, another transporter involved in doxorubicin efflux, but in our experimental conditions no nitrotyrosine residues were detectable on this protein (Fig. 3C).

**Figure 4:**
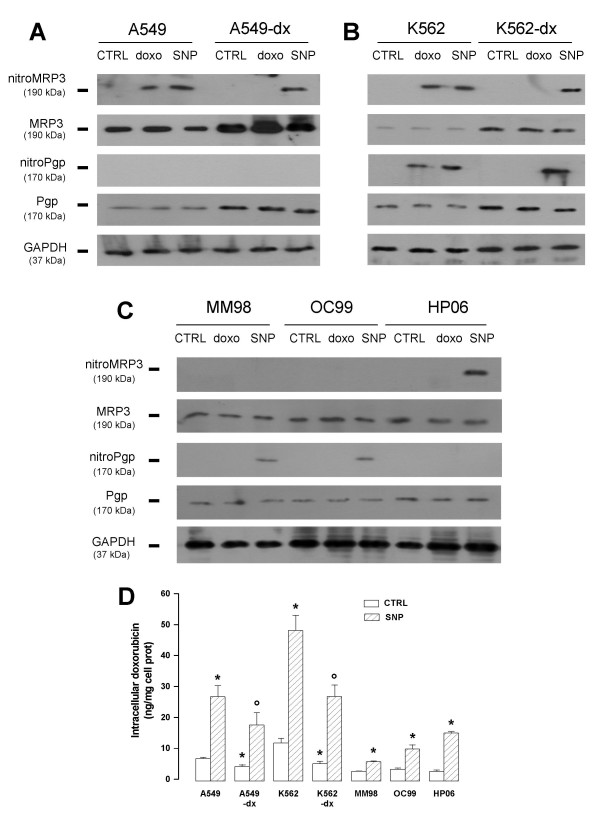
**MRP3 nitration and intracellular doxorubicin accumulation in different cell lines and primary tumours sample**. (**A, B, C**) Western blot detection of nitrated MRP3, total MRP3, nitrated Pgp, total Pgp and GAPDH. A549, A549-dx, K562, K562-dx, MM98, OC99 and HP06 cells were incubated in the absence (*CTRL*) or in the presence of 5 μmol/L doxorubicin (*doxo*) for 24 h or 100 μmol/L SNP (*SNP*) for 3 h, then lysed and subjected to immunoprecipitation with an anti-nitrotyrosine polyclonal antibody. Western blotting for MRP3 and Pgp was performed on the immunoprecipitated proteins as reported in the Methods section. An aliquot of cell lysates was directly probed with an anti-Pgp or an anti-MRP3 antibody to detect total Pgp or MRP3. The expression of the housekeeping protein GAPDH was measured as equal control loading. The results shown here are representative of two similar experiments. (**D**) Cells were incubated in the absence (*CTRL*) or in the presence of 100 μmol/L SNP, together with 5 μmol/L doxorubicin. The intracellular accumulation of the drug was measured fluorimetrically in cells (see Methods for details). The experiments were performed in triplicate and data are represented as mean ± SE (n = 3). Versus respective CTRL: * *p *< 0.02; versus A549-dx CTRL or K562-dx CTRL respectively: ° *p *< 0.02.

To exclude that the reversion of doxorubicin resistance due to the nitration of MRP3 was a phenomenon limited to colon cancer, we analyzed other doxorubicin-sensitive and doxorubicin-resistant cell lines, as well as primary tumours samples (Figure 4). Similarly to HT29 model, Pgp and MRP3 were present in the human doxorubicin-sensitive lung cancer A549 cells and increased in the doxorubicin-resistant A549-dx cells (Fig. 4A). The NO donor sodium nitroprusside (SNP) exerted a nitration of MRP3 in both A549 and A549-dx cells (Fig 4A) and increased the intracellular drug accumulation in both cell populations (Fig 4D). On the other hand, doxorubicin was able to nitrate MRP3 only in sensitive cells, where its intracellular content was significantly higher than in resistant cells. No nitration on Pgp was detected (Fig. 4A). In the chronic myelogenous cell line K562, the total amount of Pgp and MRP3 was low, whereas both transporters increased in doxorubicin-resistant K562-dx cells (Fig. 4B). However, differently from colon and lung cancer, both Pgp and MRP3 were nitrated by SNP in K562 and K562-dx cells. Again doxorubicin elicited a tyrosine nitration of these transporters only in the drug-sensitive cell line (Fig 4B). The intracellular doxorubicin accumulation was higher in K562 cells in comparison with A549 or HT29 cells, suggesting a lower activity of Pgp and MRP3 in leukaemia cells. Moreover the increase of doxorubicin content elicited by SNP was more pronounced in K562 cells, probably because of the nitration of both Pgp and MRP3 in these cells (Fig 4D). We also examined three primary tumours samples: MM98 and OC98 malignant mesothelioma cells, and HP06 metastatic breast cancer cells. All samples expressed Pgp and MRP3, and basally accumulated very low amounts of doxorubicin, suggesting that they were constitutively resistant to doxorubicin (Fig 4C and 4D). As expected, doxorubicin failed to nitrate Pgp or MRP3; however also in these models the NO donor SNP induced a tyrosine nitration of the ABC-transporters (respectively Pgp in MM98 and OC99 cells, MRP3 in HP06 cells; Fig 4C) and increased the intracellular doxorubicin accumulation (Fig 4D).

### iNOS activity is necessary to mediate the pro-immunogenic effects of doxorubicin

**Figure 5:**
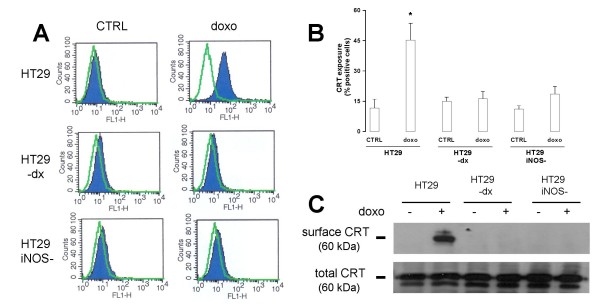
**Detection of surface calreticulin in HT29, HT29-dx and HT29 *iNOS*^- ^cells**. Cells were grown in absence (*CTRL*) or presence of 5 μmol/L doxorubicin (*doxo*) for 24 h and subjected to the following investigations. (**A**, **B**) FACS analysis of surface CRT. Negative controls, with non-immune isotypic antibodies, are shown in **A **by green outline. The figures shown here are representative of three similar experiments, performed in triplicate. In panel **B **the percentage of cells positive for surface calreticulin is represented as mean ± SE in all experiments. Versus HT29 CTRL: * *p *< 0.005. (**C**) Western blot detection of surface CRT and total CRT. The measurement of CRT associated with the plasma membrane was performed in a biotinylation assay as described in the Methods section. Under the same experimental conditions, an aliquot of cells was lysed and ultracentrifuged to detect the total CRT amount. The results shown here are representative of two similar experiments.

Flow cytometry analysis showed that the fraction of tumour cells with externalized CRT was low in all the cell lines (Fig. 5A, 5B) and was significantly increased after doxorubicin treatment in the HT29 cells (Fig. 5A, 5B). On the contrary, the CRT exposure on the membrane of HT29-dx and HT29 *iNOS*^- ^cells was not changed by doxorubicin. The results obtained in the biotinylation assays, which examined only the CRT fraction present on the cell surface, confirmed the flow cytometry results (Fig. 5C). The amount of CRT in the total cell lysate was similar in all the three cell lines and was not affected by doxorubicin (Fig. 5C).

**Figure 6:**
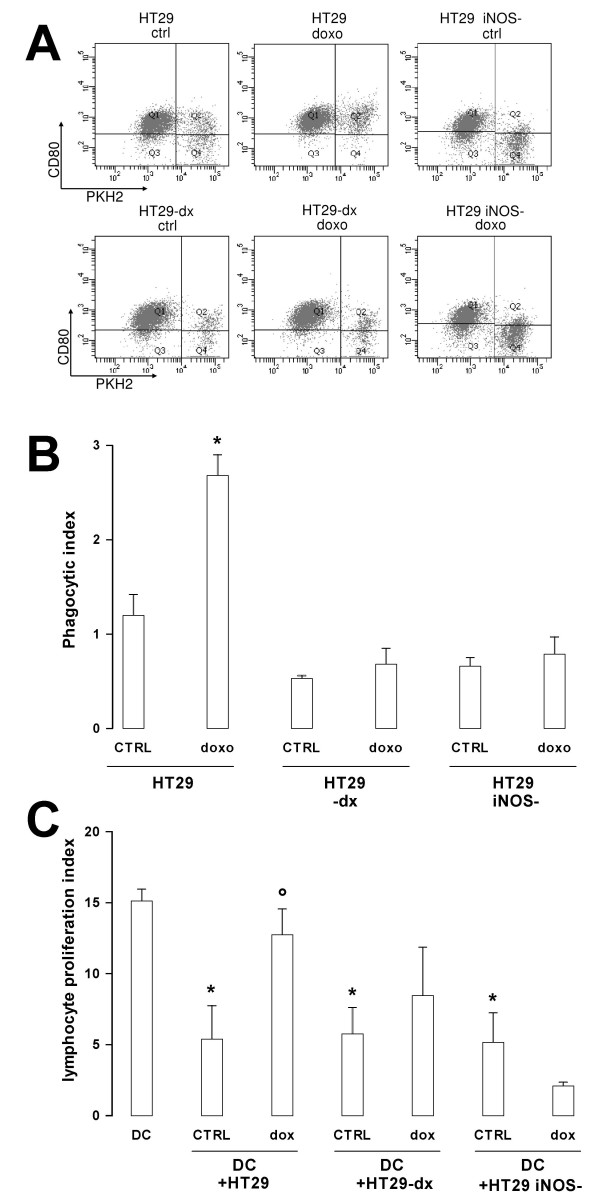
**Phagocytic and alloantingenic presenting activity of DCs loaded with HT29, HT29-dx and HT29 *iNOS*^- ^cells**. After a 24 h incubation in the absence (*CTRL*) or in the presence of 5 μmol/L doxorubicin (*doxo*), the tumour cells were green-stained with PKH2-FITC and subjected to the phagocytosis assay as described in the Methods section. The dot plot analysis of a phagocytosis assay, representative of three similar experiments performed in duplicate, is shown in panel **A**. In panel **B **the phagocytic index is represented as mean ± SE of all the experiments. Versus HT29 CTRL: * *p *< 0.02. **C**. DCs unloaded (*DC*), loaded with untreated tumour cells (*CTRL*) or loaded with doxorubicin-treated tumour cells (*dox*), in the same experimental conditions of panel **B**, were treated with mitomycin C and co-cultured with PBMCs, containing the allogenic lymphocytes. At day 4 cells were labelled with [methyl-^3^H]deoxythymidine and the *cpm *were evaluated in a β-counter. The [methyl-^3^H]deoxythymidine incorporation of lymphocytes alone were 1985.33 ± 298.19 *cpm*. The Proliferation Index of each culture was calculated as detailed in the Methods section. The experiments were performed in triplicate and the results are expressed as mean ± SE from two experiments with lymphocytes from different donors. Versus DC: * *p *< 0.01; versus HT29 CTRL: ° *p *< 0.05.

The rate of tumour cells phagocytosis was positively correlated with the levels of exposed calreticulin in that it was increased by doxorubicin in HT29 cells, but not in HT29-dx and HT29 *iNOS*^-^cells (Fig. 6A, 6B). Functional maturation of DCs followed the same pattern, with increased alloantigen presentation by DC that had phagocyted doxorubicin-treated HT29 (Fig 6C). It is noteworthy in this context that doxorubicin-treatment overcame the inhibition induced on DC exposed to the untreated tumour. On the contrary, experimental conditions that failed to increase the tumour cells uptake (Fig. 6A, 6B) did not reverse this inhibition. No activation was observed in lymphocytes cultured with HT29, HT29-dx or HT29 *iNOS*^- ^tumour cells in the absence of DCs (not shown).

### The chemoresistance towards doxorubicin is reversed by increasing the NO levels in HT29 *iNOS*^- ^cells

**Figure 7:**
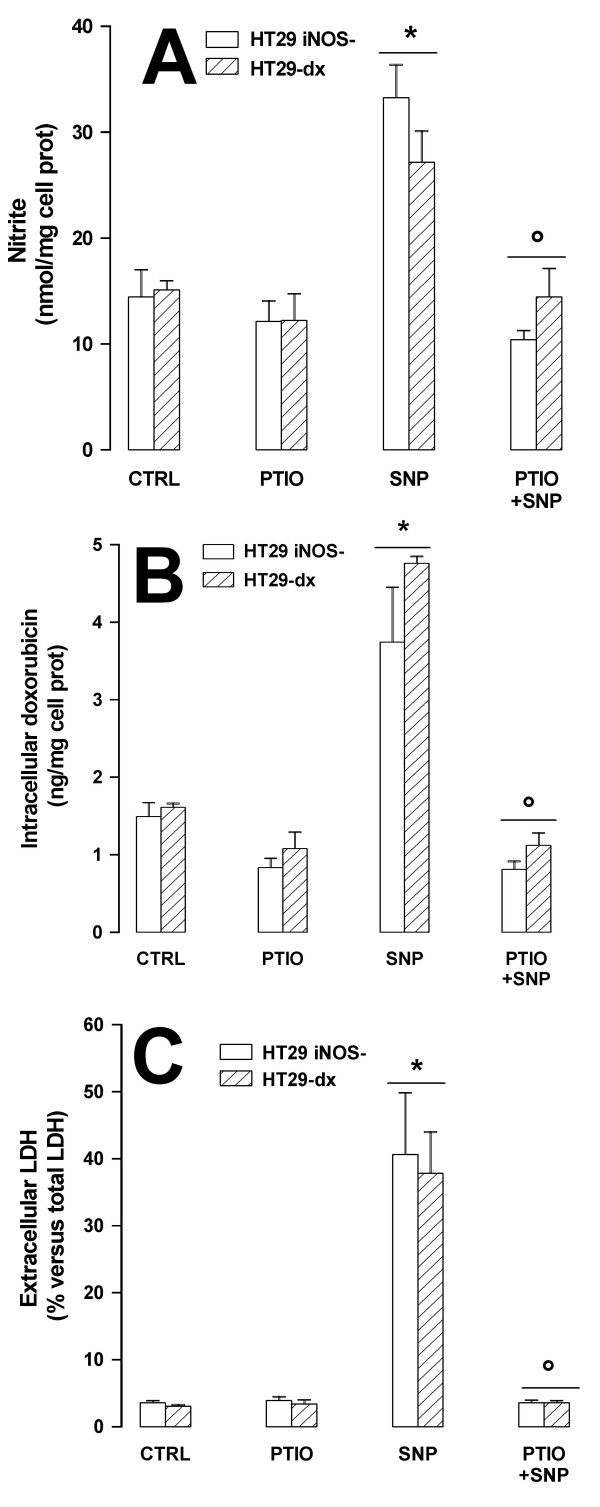
**Effect of SNP and PTIO on nitrite levels and doxorubicin toxicity in HT29 *iNOS*^- ^cells**. HT29 *iNOS*^- ^cells and HT29-dx cells were incubated for 3 h in the absence (*CTRL*) or presence of PTIO (100 μmol/L), SNP (100 μmol/L) or both (*PTIO+SNP*), then subjected to the following investigations. (**A**) Nitrite levels in extracellular medium were measured by Griess method as reported in the Methods section. Measurements were done in triplicate and data are represented as mean ± SE (n = 3). Versus CTRL: * *p *< 0.05; versus SNP: ° *p *< 0.05. (**B**). Intracellular accumulation of doxorubicin was measured fluorimetrically in cells incubated in the absence (*CTRL*) or in the presence of PTIO, SNP and PTIO+SNP, together with 5 μmol/L doxorubicin (see Methods section). The experiments were performed in triplicate and data are represented as mean ± SE (n = 3). Versus CTRL: * *p *< 0.002; versus SNP: ° *p *< 0.01. (**C**) The release of LDH in the cell culture medium was measured in triplicate under the same experimental conditions of point **B**. In the absence of doxorubicin, neither PTIO nor SNP significantly changed the extracellular activity of LDH towards control cells (data not shown). Data are represented as mean ± SE (n = 4). Versus CTRL: * *p *< 0.02; versus SNP: ° *p *< 0.01.

To provide a further confirmation of the correlation between NO levels and chemoresistance to doxorubicin, we modulated the level of NO in HT29 *iNOS*^- ^cells by adding the NO-donor SNP and by removing NO with 2-phenyl-4,4,5,5,-tetramethylimidazoline-1-oxyl 3-oxide (PTIO), chosen as a NO scavenger. After 3 h SNP increased the nitrite levels in the culture medium of HT29 *iNOS*^- ^cells, and such an increase was effectively abolished by PTIO (Fig. 7A). Cells were checked for the expression of eNOS, nNOS and iNOS proteins: neither PTIO nor SNP changed the eNOS and nNOS levels when compared to the untreated HT29 *iNOS*^-^, and iNOS remained absent under each experimental condition (data not shown). As far as the doxorubicin content and toxicity are concerned, the intracellular drug accumulation and the extracellular LDH release were significantly increased in the presence of SNP, whereas the combination of PTIO and SNP reduced both the drug content and toxicity to the control levels (Fig. 7B, 7C). PTIO alone also slightly diminished the intracellular doxorubicin accumulation. In drug-resistant HT29-dx cells PTIO and SNP had the same effects on nitrite synthesis (Fig 7A), intracellular doxorubicin accumulation (Fig 7B) and cytotoxicity (Fig 7C) than in HT29 *iNOS*^- ^cells.

### Restoration of NO levels is *per se *sufficient to elicit the calreticulin translocation and the phagocytosis of HT29 *iNOS*^- ^cells

**Figure 8:**
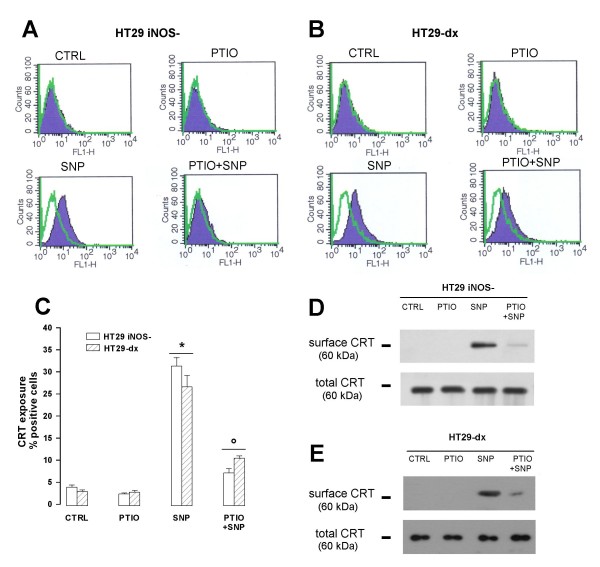
**Effect of SNP and PTIO on calreticulin exposure in HT29 *iNOS*^- ^cells**. HT29 *iNOS*^- ^cells and HT29-dx cells were incubated for 3 h in the absence (*CTRL*) or in the presence of PTIO (100 μmol/L), SNP (100 μmol/L) or both (*SNP+PTIO*) for 3 h, and then the surface CRT was investigated by the following procedures. (**A, B, C**) FACS analysis of surface CRT. The negative controls, with non-immune isotypic antibodies, are shown by green outline in panel **A **and **B**. The figures shown here are representative of three similar experiments, done in triplicate. In panel **C **the percentage of cells positive for surface CRT is represented as mean ± SE in all experiments. Versus CTRL: * *p *< 0.05; versus SNP: ° *p *< 0.05. (**D, E**) Western blot detection of surface CRT and total CRT in HT29 *iNOS*^- ^cells and HT29-dx cells after a biotinylation assay (see Methods for details). Total CRT was detected in an aliquot of cells incubated under the same experimental conditions of the biotinylation assay, subjected to ultracentifugation and Western blot analysis. The results shown here are representative of two similar experiments, with superimposable results.

**Figure 9:**
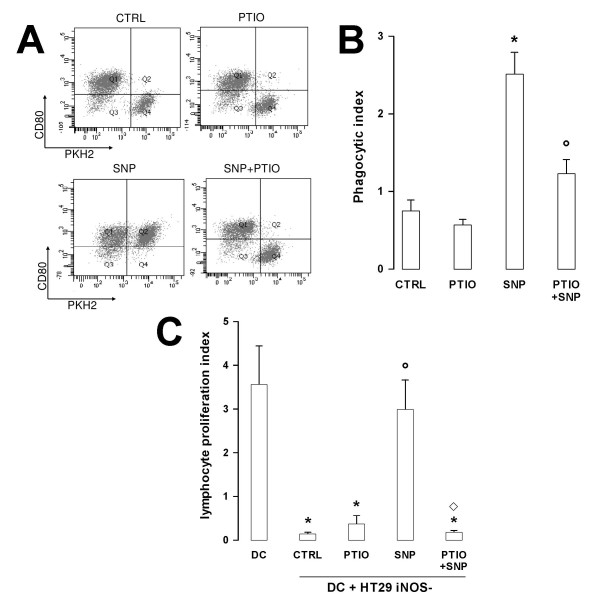
**Phagocytic and alloantingenic presenting activity of DCs loaded with HT29 *iNOS*^- ^cells in the presence of SNP and PTIO**. Cells were incubated for 3 h in fresh medium (*CTRL*), PTIO (100 μmol/L), SNP (100 μmol/L) or both (*SNP+PTIO*), then stained with PKH2-FITC and co-incubated with DCs for the phagocytosis assay, as described in the Methods section. The dot plot analysis of a phagocytosis assay, representative of three similar experiments performed in duplicate, is shown in panel **A**. In panel **B **the phagocytic index is represented as mean ± SE of all the experiments. Versus CTRL: * *p *< 0.05; versus SNP: ° *p *< 0.05. **C**. Unloaded DCs (*DC*) or DCs loaded with HT29 *iNOS*^- ^cells in the same experimental conditions of panel **B **were inhibited by mitomycin and the DNA synthesis of co-cultured allogenic lymphocytes was measured as reported in the legend of Figure 6. The Proliferation Index was measured as described in the Methods section. The [methyl-^3^H]deoxythymidine incorporation of lymphocytes alone were 899.33 ± 96.56. The experiments were performed in triplicate and the results are expressed as mean ± SE from four experiments with lymphocytes from different donors. Versus DC: * *p *< 0.02; versus CTRL: ° *p *< 0.02; versus SNP: ° *p *< 0.05.

SNP *per se *was able to elicit the translocation of CRT on the plasma membrane of HT29 *iNOS*^- ^cells, as demonstrated by flow cytometry analysis (Fig. 8A, 8C) and biotinylation assays (Fig. 8D). Again the effect of the NO donor was reduced by PTIO (Fig. 8). Neither SNP nor PTIO changed the amount of total CRT in comparison with the untreated HT29 *iNOS*^- ^cells (Fig. 8D). Superimposable effects on CRT translocation were exerted by SNP and PTIO on the drug-resistant HT29-dx cells (Fig 8B, 8C and 8E). NO levels played a role also in the uptake of tumour cells by DCs and in the further DCs-elicited lymphocytes activation: indeed the presence of SNP was sufficient to increase the phagocytosis of HT29 *iNOS*^- ^cells by DCs (Fig 9A and 9B) and the subsequent expansion of lymphocytes in response to DCs (Fig 9C), whereas the addition of PTIO reversed these effects.

## Discussion

Exerting a strong cytotoxic effect and enhancing the host immune response against tumour cells are two goals of an ideal anti-cancer therapy, which should improve the efficacy of the conventional treatments, like chemotherapy and radiotherapy, and improve the patients prognosis [13].

The anthracyclines family, in particular doxorubicin, are among the first therapeutic choices in solid tumours and are the only known chemotherapeutics agents capable of not only reduce the tumour cell mass, but also confer long term protection against recurrent tumour [3,4,14], an effect mediated by dendritic cells (DCs). DCs capture tumour cells and present to T-cells the antigen epitopes on class I MHC, thus cross-priming CD8 T lymphocytes. Antigen capturing is property of immature DC (*i*DC), whereas antigen presentation is a characteristic of the lymph node migrating mature DC. The context in which the tumour is captured by *i*DC dictates its immunogenicity, a paramount role being played by molecules induced by death agents. Calreticulin has been shown to be translocated on plasma membrane by anthracyclines and to trigger tumour cells uptake by *i*DC [3,4,14]. Moreover doxorubicin may exert a direct cytotoxic effect on transformed cells with several mechanisms, e.g. by intercalating amongst DNA bases, impairing the chromatin folding, inhibiting the topoisomerase II activity and generating reactive oxygen species [15]. At least part of the cytotoxicity of this anthracycline is dependent on the synthesis of NO [7], which can exert a pro-apoptotic effect on tumour cells [9,16].

This work was aimed at investigating whether the cytotoxic and pro-immunogenic effects of doxorubicin rely on the same mechanism, i.e. the synthesis of NO elicited by the drug in tumour cells, through the induction of *iNOS *gene [5,7]. We have previously observed that in drug-resistant cancer cells doxorubicin fails to increase NO levels [5]; we thus wondered whether the inability of tumour cells to increase the NO production in response to the drug, besides determining a chemoresistant phenotype, also results in an immunoresistant status.

In order to investigate the importance of NO in chemo- and immunoresistance, from the doxorubicin-sensitive HT29 cells we developed the HT29 *iNOS*^- ^cell line, which was deprived of iNOS protein by a specific and stable RNA interfering procedure. In this cell population doxorubicin did not increase the NOS activity and the nitrite levels, as a consequence of the *iNOS *knocking-down. Regarding NO synthesis, HT29 *iNOS*^- ^cells became functionally equivalent to the HT29-dx cell line, a drug-resistant population, in which doxorubicin is actively extruded by Pgp and MRP3 membrane transporters. In HT29-dx cells the drug has not sufficient time to accumulate and induce the transcription of *iNOS *gene, and this results in the lack of NO increase after doxorubicin exposure [5]. The characterization of HT29, HT29-dx and HT29 *iNOS*^- ^cells is summarized in table 1.

**Table 1:** Characterization of HT29, HT29-dx and HT29 *iNOS*^- ^cells

Parameters	HT29 cells	HT29-dx cells	HT29 *iNOS*^- ^cells
**Doxorubicin-induced** **expression of iNOS protein**	Increased	Absent	Absent
**Doxorubicin-induced** **expression of iNOS mRNA**	Increased	Absent	Absent
**Doxorubicin-induced** **NOS activity**	High	Low	Low
**Intracellular doxorubicin** **Accumulation**	High	Low	Low
**LDH release after** **Doxorubicin**	High	Low	Low
**MRP3 expression**	Low	High	Low
**Doxorubicin-induced** **MRP3 nitration**	Present	Absent	Absent
**Pgp expression**	Low	High	Low
**Doxorubicin-induced** **Pgp nitration**	Absent	Absent	Absent
**Doxorubicin-induced** **CRT translocation**	Increased	Absent	Absent
**Doxorubicin-induced** **DC activation**	Increased	Absent	Absent

Real Time-PCR and Western blotting experiments showed the absence of the *iNOS *mRNA and protein in HT29 *iNOS*^- ^cells. On the other hand, we observed in this cell line an increase of eNOS and nNOS isoforms, suggesting that cells react to the absence of iNOS with an overexpression of the other two NOS isoforms. Such a crosstalk among eNOS, nNOS and iNOS has been already described: indeed it has been reported that when the expression of iNOS is high, the levels of the constitutive NOS isoforms are down-regulated, and vice versa [17-20]. This event may be due to a feedback inhibition exerted by NO itself on constitutive NOS protein levels: for example in endothelial cells the shear stress enhances the transcription of eNOS gene in a NF-kB dependent way, but the NO donor DPTA-NO, which nitrosylates NF-kB on p50 subunit, prevents the NF-kB translocation and the eNOS increase [21]. Doxorubicin is known to activate NF-kB [9] and to induce the expression of iNOS [8]. It is conceivable that the huge amounts of NO produced in response to doxorubicin could inhibit the further activation of NF-kB and limit the expression of eNOS in HT29 cells; on the contrary, in HT29 *iNOS*^- ^cells doxorubicin is free to activate NF-kB, but not to induce iNOS gene. Since the NO-mediated inhibition on NF-kB is lacking in HT29 *iNOS*^- ^cells, NF-kB may translocate into the nucleus and increase the transcription of eNOS gene in these cells. This mechanism might explain why eNOS expression is increased in HT29 *iNOS*^- ^cells exposed to doxorubicin. Few data exist on the reciprocal regulation of nNOS and iNOS [20], but a similar mechanism may be hypothesized to explain also the increase nNOS expression in HT29 cells silenced for iNOS. Although in our experimental conditions eNOS and nNOS proteins were up-regulated, the global NOS activity in HT29 *iNOS*^- ^cells was not higher than in control HT29 cells. This is not surprising, because eNOS and nNOS proteins have a very low basal activity and are both Ca^2+^/calmodulin-dependent; therefore they reach a maximal activity only when Ca^2+ ^is increased within cells [22], which is not the case of untreated HT29 *iNOS*^- ^cells. Thus it seems conceivable that eNOS and nNOS, although over-expressed, were poorly active in HT29 *iNOS*^- ^cells.

To evaluate whether the lack of NO, independently from the underlying molecular mechanism (increased doxorubicin efflux, as occurs in HT29-dx cells, or absence of iNOS enzyme, as seen in HT29 *iNOS*^- ^cells), was important for chemo- and immunoresistance, we examined the doxorubicin accumulation and cytotoxicity in wild-type HT29 cells, in HT29-dx cells and in HT29 *iNOS*^- ^cells. The three cell lines markedly differed in the amount of drug efflux pumps: both Pgp and MRP3 were low in the parental HT29 and in the *iNOS*-silenced cells derived from HT29, and were over-expressed in HT29-dx cells. However, HT29 *iNOS*^- ^had the same behaviour of HT29-dx cells in accumulating low amount of doxorubicin and being poorly damaged by the drug. This result suggests that NO regulates the cell resistance to doxorubicin, independently from the absolute amount of the drug efflux pumps.

We hypothesized that the low intracellular accumulation of doxorubicin observed in HT29 *iNOS*^- ^cells was a consequence of an increased activity of the drug efflux trough Pgp and MRP3, as occurs in HT29-dx cells. Indeed NO can enhance doxorubicin accumulation by nitrating tyrosine residues of the MRP3 pump [5,9,10]. Because HT29 *iNOS*^- ^cells had the same amount of MRP3 and Pgp of HT29 cells, we checked whether a different amount in nitrated tyrosine residues of MRP3, which is related to a different activity of the pump [5,9,10], may explain the differences in doxorubicin accumulation between these two cells lines. Indeed, doxorubicin did increase the nitration of MRP3 pump only in HT29 cells, in accordance with the higher NO levels observed after the exposure to the drug; on the contrary, HT29 *iNOS*^- ^cell line did not show any nitrated tyrosine residues on MRP3, as well as HT29-dx cells. It is conceivable that NO exerts a restraint on MRP3 activity, therefore the lack of NO synthesis allows the MRP3 to freely pump doxorubicin out, limiting its intracellular accumulation and toxicity, and inducing a chemoresistant phenotype. Although Pgp is a second crucial transporter for doxorubicin, in our experimental conditions no nitration has been detected. The protein amounts and localization, the tyrosine accessibility and number, the presence of particular amino acidic sequences around the tyrosines are considered critical factors in determining the nitration of a protein [23]. The lower abundance of Pgp respect to MRP3 or the lower number of accessible tyrosine on Pgp may account for the absence of nitrotyrosine on Pgp in HT29 cells.

To our knowledge, no epidemiological data exist about the frequency of nitration of MRP3 in colon cancer. However, the nitration of cell proteins, such as tubulin, actin and β-catenin, has been reported in inflammatory bowel diseases [24] and in colon cancers [25,26], where a sustained NO synthesis (mainly due to the activation of iNOS) occurred. MRP3 is particularly abundant in colon tumour specimens [27,28]; therefore we hypothesize that its nitration, e.g. following the activation of iNOS by inflammatory stimuli of tumour microenvironment or by doxorubicin, is not so rare in human colon cancers. Our work shows that the nitration of the drug efflux transporters occurs in cell lines other than colon and is detectable also in primary tumour samples. In each tissue a different pathway of nitration of Pgp or MRP3 occurs: the discrepancies may depend on the relative amounts of each transporter or on the accessibility of the target tyrosines, which may vary among the isoforms of Pgp and MRP3 present in different tissues. However, independently from which transporter is nitrated, the nitration of either Pgp or MRP3 leads to a decrease of the drug efflux and revert the resistance to doxorubicin.

When activated by doxorubicin, iNOS can produce micromolar amounts of NO [29], which at such high levels may act as a anti-cancer agent by impairing the aerobic mitochondrial metabolism, inhibiting the ribonucleotide reductase enzyme and directly damaging DNA via a nitrosative and oxidative stress [16,29]. NO is also considered an immuno-modulatory agent at tumour sites, where it can differently regulate the activity of the host immune system [30]. Starting from the observation that part of the doxorubicin anti-cancer effects relies on the activation of immune system cells, through CRT exposure and DC recruitment, we investigated which role NO may play in these events.

Doxorubicin induced CRT exposure on the cell membrane in HT29 cells but not in HT29-dx and in HT29 *iNOS*^- ^cells. In keeping with the CRT surface levels, doxorubicin induced the tumour cells phagocytosis by DCs in the chemosensitive HT29 cells, but not in chemoresistant HT29-dx cells and HT29 *iNOS*^- ^cells. The translocation of calreticulin and the subsequent tumor cells uptake by immature DCs are the first steps along a doxorubicin-induced DCs stimulatory pathway. Indeed, when chemosensitive HT29 cells were exposed to the drug, they were engulfed with higher efficiency by DCs, with a consequent increase of alloantigen presenting activity by DCs. On the contrary, doxorubicin treatment of HT29-dx and HT29-*iNOS*^- ^cells failed to induce CRT translocation, did not change the uptake of the tumour and had no effect on the alloantigen presenting activity of DCs. In the absence of DCs, tumour cells alone were incompetent to trigger lymphocytes proliferation, despite the high levels of CRT, as occurred in HT29 cells treated with doxorubicin. These results suggest that doxorubicin requires the presence of local DCs, which actively phagocyte CRT-positive tumor cells and bona fide cross-present the tumor antigens [4], to recruit host lymphocytes against the tumor and exert a significant pro-immunogenic effect. Of interest in our study is the description of the ability of doxorubicin to reverse the immunosuppression exerted by the HT29 cells on functional maturation of DCs, an event in keeping with our previous findings on a gastric cell line [31], a renal carcinoma [32] and a prostate carcinoma cell line [33].

Despite significant changes in the CRT exposed on the plasma membrane we did not observe any variation in total CRT. This result suggests that the change in CRT levels on the cell surface should be due to a translocation from ER to the plasma membrane, rather than to a *de novo *synthesis of the protein. Indeed most CRT usually resides in the ER and is absent on the plasma membrane of untreated cells, as we detected in control HT29, HT29-dx and HT29 *iNOS*^- ^cells. It has been suggested that also small variations in the CRT amount in plasma membrane may be sensed as an effective "eat me signal" and produce dramatic effects on DCs recruitment [4].

At the light of our results, we may hypothesize that the lack of CRT exposure following doxorubicin incubation in HT29-dx and HT29 *iNOS*^- ^cells is mainly due to the low intracellular drug content, which is insufficient to elicit the translocation of CRT; on the other hand, we cannot exclude that in these cells the lack of CRT exposure may be a consequence of the insufficient synthesis of NO, which has already been shown to enhance the translocation of proteins from cytosol to plasma membrane [12].

To clarify whether NO mediates the doxorubicin-induced exposure of CRT, we changed the NO levels in the doxorubicin-resistant and *iNOS*-deprived cells: if NO is critical for the doxorubicin immunogenic effects, we should be able to observe the translocation of CRT followed by the phagocytosis of HT29 *iNOS*^- ^cells, simply by increasing the NO level. To this purpose we chose the NO-donor SNP, which releases huge amounts of NO after interacting with reducing agents, such as intracellular thiols [16]. SNP increased the nitrite levels, the intracellular accumulation and the toxicity of doxorubicin in HT29 *iNOS*^- ^cells. These effects were prevented by the co-incubation with an equimolar concentration of the NO-scavenger PTIO. Most importantly, SNP alone up-regulated the CRT exposure on the cell membrane of the *iNOS *deprived cells and enhanced the cells uptake by DCs, as well as the activation of lymphocytes recognizing the alloantigen presented by DCs. Taken as a whole, these results show that in the chemoresistant HT29 *iNOS*^- ^cell line a NO donor reproduces the effects exerted by doxorubicin in the chemosensitive HT29 cells. PTIO succeeds in reducing the CRT exposure and the cell phagocytosis induced by SNP, transforming again the immunogenic phenotype of HT29 *iNOS*^- ^cells into non-immunogenic phenotype. We obtained the same effects on doxorubicin accumulation and CRT exposure in HT29-dx cells. These results suggest that NO levels are crucial in modulating the immune- and chemoresistance in human colon cancer cells. The mechanism by which NO mediates the CRT translocation is currently under investigation in our laboratory: preliminary data suggest an involvement of the guanylate cyclase/protein kinase G pathway and of the actin fibers cytoskeleton remodelling in such a translocation.

## Conclusion

To our knowledge, this is the first work showing that chemo- and immunoresistance may be strictly associated in cancer cells. Our results suggest that NO is a mediator for these events and that through a common strategy (augmenting NO levels), both chemo- and immunoresistance can be reversed. Notably NO-releasing drugs are widely used in the therapy of cardiovascular diseases and NO-donors are objects of intensive investigations as potential anticancer drugs, as single cytotoxic agents as well as in combination with standard radio- and chemotherapy [34,35]. Our findings that these compounds are involved in both the anticancer and pro-immunogenic effects of anthracyclines may pave the way to their future application in the therapy of solid tumours.

## Methods

### Materials

Foetal bovine serum (FBS), penicillin-streptomycin (PS) and RPMI 1640 were supplied by Sigma Chemical Co (St. Louis, MO), plasticware for cell culture was from Falcon (BD Biosciences, Bedford, MA). Electrophoresis reagents were obtained from Bio-Rad Laboratories (Hercules, CA), the protein content of cell monolayers and cell lysates was assessed with the bicinchoninic acid kit from Sigma Chemical Co. When not otherwise specified, all the other reagents were purchased from Sigma Chemical Co.

### Cells

Human colon cancer cells (HT29 cell line, provided by Istituto Zooprofilattico Sperimentale "Bruno Umbertini", Brescia, Italy) were cultured in RPMI 1640 medium supplemented with 10% FBS, 1% PS, and 1% L-glutamine and maintained in a humidified atmosphere at 37°C and 5% CO_2_.

A subpopulation of HT29 cells, named HT29-dx, was created according to the methods of Riganti et al. [5] and subsequently cultured in RPMI 1640 medium containing 150 nmol/L doxorubicin as "maintenance dose". HT29-dx cells exhibited a higher amount of Pgp and MRP3, a significantly lower intracellular accumulation of doxorubicin after a drug bolus (5 μmol/L) and were more resistant to the drug's toxic effects, as previously described [5].

Human doxorubicin-sensitive lung cancer A549 cells (purchased from Istituto Zooprofilattico Sperimentale "Bruno Umbertini", Brescia, Italy) and human doxorubicin-resistant lung cancer A549-dx cells (selected from the parental A549 cells after 30 passages in medium containing 50 nM doxorubicin) were grown in HAM'S F12 medium, with 10% FBS, 1% PS, and 1% L-glutamine. Human doxorubicin-sensitive chronic myelogenous leukaemia cells K562 (from Istituto Zooprofilattico Sperimentale "Bruno Umbertini") and human doxorubicin-resistant chronic myelogenous leukaemia cells K562-dx (obtained after 30 passages of parental K562 in medium containing 25 pM doxorubicin) were cultured in RPMI 1640 medium, in the presence of 10% FBS, 1% PS, and 1% L-glutamine. MM98 and OC99 cells, two primary malignant mesothelioma cell cultures established from the pleural effusion of two patients with histologically confirmed malignant mesotelioma, were already described [11]. HP06 cells, derived from the peritoneal metastasis of a female patient with an invasive breast cancer, was a gift from Prof. Anna Sapino (Department of Biomedical Sciences and Oncology, University of Turin, Italy); cells were grown in DMEM medium with 10% FBS, 1% PS, and 1% L-glutamine and were used at passages 2-4.

### iNOS siRNA Transfection

200,000 cells were plated in 35-mm-diameter Petri dishes and cultured in RPMI 1640 medium. After 24 h, the medium was removed and the cells were incubated for 6 h with 250 μL ESCORT DNA mix (Sigma Chemical Co.), containing 15 μL ESCORT reagent and 1 μg pLKO.1-puro vector, in which the iNOS MISSION shRNA (code 10270809MN, Sigma Chemical Co.) was subcloned. Then the medium was removed and 2 mL RPMI 1640 (with 20% FBS and 1% PS) were added. After 48 h from the transfection, the cells were cultured in RPMI 1640 containing 2 nmol/L puromycin, to select the clones silenced for *iNOS *gene. To verify that the silencing was successful, we checked the *iNOS *mRNA levels by Real time-PCR, the iNOS protein expression by Western blotting and the NO synthesis as reported below. To ascertain the specificity of the silencing experimental procedure, an aliquot of HT29 cells was treated with 1 μg pLKO.1-puro vector containing a scrambled non-targeting 20-25 nucleotide shRNA, instead of iNOS shRNA, and the iNOS expression evaluated in this cell population was used as a negative control.

### Nitrite Production

Confluent monolayers in 35-mm-diameter Petri dishes were incubated in fresh medium under the experimental conditions indicated in Results. Nitrite production was measured by adding 0.15 mL of cell culture medium to 0.15 mL of Griess reagent in a 96-well plate, and after a 10 min incubation at 37°C in the dark, the absorbance was detected at 540 nm with a Packard EL340 microplate reader (Bio-Tek Instruments, Winooski, VT). A blank was prepared for each experiment in the absence of cells, and its absorbance was subtracted from that obtained in the presence of cells. Nitrite concentration was expressed as nanomoles of nitrite/mg cell protein.

### Measurement of NOS Activity

To measure NOS activity, the procedure described in Ghigo et al. [36] was followed. The cells were detached with 0.05% v/v trypsin, washed with PBS, re-suspended at the concentration of 100 μg cell proteins in 0.6 mL of 20 mmol/L Hepes buffer (pH 7.2) and sonicated with one 10 s burst, using a Labsonic Sonicator (Hielscher, Teltow, Germany). 15 μL of lysate was withdrawn to asses the protein content. The protease inhibitors pepstatin (75 μmol/L) and leupeptin (20 μmol/L) were added to the cell lysate, which was then mixed with the following reagents in a 500 μL final volume: 0.2 mmol/L NADP^+^, 360 μmol/L L-arginine, 2 μmol/L tetrahydrobiopterin, 0.3 mmol/L CaCl_2_, 0.2 mmol/L dithiothreitol, 1.8 mmol/L MgCl_2_, 0.17 mmol/L glucose 6-phosphate, and 40 mU/mL glucose 6-phosphate dehydrogenase (G6PD; from *Saccaromyces cerevisiae; *EC 1.1.1.49). The NOS/G6PD reaction mixture described above was incubated at 37°C for 3 h, then heated at 100°C for 5 min to inactivate G6PD. To oxidize the remaining NADPH, which might interfere with the subsequent Griess reaction, the mixture was incubated with 10 mU/mL L-lactate dehydrogenase (from pig muscle; EC 1.1.1.27) and 300 μmol/L sodium pyruvate at 37°C for 5 min. The nitrite production was measured using the Griess reagent as described. Blanks were prepared by replacing the cell lysate with Hepes solution, in the NOS/G6PD reaction mix, which was subjected to the same experimental procedure used for samples containing lysate. The NOS activity was expressed as nanomoles nitrites/min/mg protein.

### Real Time-PCR

Total RNA was obtained as previously described [37]. 5 μg of RNA was reverse-transcribed by 200 U of M-MLV reverse transcriptase (Invitrogen, Milan, Italy), in the presence of 40 U/μL RNAseOUT (Invitrogen). Quantitative Real Time-PCR was carried out using IQ™ SYBR Green Supermix (Bio-Rad), according to the manufacturer's instructions. The same cDNA preparation was used for the quantitation of iNOS and GAPDH, used as an housekeeping gene. The sequences of the primers of *iNOS *were: 5'-ACAACAAATTCAGGTACGCTGTG-3', 5'-TCTGATCAATGTCATGAGCAAAGG-3'; the sequences of the *GAPDH *primers were: 5'-GAAGGTGAAGGTCGGAGT-3', 5'-CATGGTGGAATCATATTGGAA-3'. PCR amplification for iNOS was: 1 cycle of denaturation at 94°C for 3 min, 45 cycles of denaturation at 94°C for 30 s, annealing at 58°C for 30 s and synthesis at 72°C for 30 s; for GAPDH: 1 cycle of denaturation at 94°C for 3 min, 40 cycles of denaturation at 94°C for 30 s, annealing at 58°C for 30 s and synthesis at 72°C for 30 s. The relative quantitation of each sample was performed comparing the Pgp PCR product with the GAPDH product, using the Bio-Rad Software Gene Expression Quantitation (Bio-Rad).

### Intracellular Doxorubicin Accumulation

Cells were grown in 35-mm-diameter Petri dishes and incubated as reported in the Results section in RPMI 1640 medium containing 5 μmol/L doxorubicin. Then cells were washed twice with PBS and detached with trypsin/EDTA. The cells were centrifuged for 30 s at 13,000 × *g*, re-suspended in 1 mL of a 1:1 mixture of ethanol/0.3 N HCl and sonicated. The protein content of the cell lysates was measured and the amount of intracellular doxorubicin was detected using a PerkinElmer LS-5 spectrofluorimeter (PerkinElmer, Waltham, MA). Excitation and emission wavelengths were 475 and 553 nm, respectively. A blank was prepared in the absence of cells in each set of experiments and its fluorescence was subtracted from that measured in each sample. Fluorescence was converted in ng doxorubicin/mg cells proteins using a calibration curve prepared previously.

### Extracellular LDH Activity

To verify the cytotoxic effect of doxorubicin, the extracellular medium was centrifuged at 12,000 × *g *for 15 min to pellet cellular debris, whereas cells were washed with fresh medium, detached with trypsin/EDTA, re-suspended in 0.2 ml of 82.3 mmol/L triethanolamine phosphate-HCl (pH 7.6) and sonicated on ice with two 10 s bursts. LDH activity was measured in the extracellular medium and in the cell lysate: 50 μl of supernatant from extracellular medium or 5 μL of cell lysate were incubated at 37°C with 5 mmol/L NADH. The reaction was started by adding 20 mmol/L pyruvic acid and was followed for 6 min, measuring absorbance at 340 nm with Packard EL340 microplate reader (Bio-Tek Instruments). The reaction kinetics was linear throughout the time of measurement. Both intracellular and extracellular enzyme activity was expressed in μmol NADH oxidized/min/dish, then extracellular LDH activity was calculated as percentage of the total LDH activity in the dish.

### Flow Cytometry Analysis

Cells grown at confluence on 35-mm-diameter Petri dishes were washed twice with PBS, detached with Cell Dissociation Solution (Sigma), rinsed with 1 mL of 0.25% w/v PBS-bovine serum albumin (BSA) and centrifuged at 10,000 × *g *for 5 min. The cells were incubated for 45 min at 4°C with an anti-CRT rabbit polyclonal antibody (catalogue number PA3-900, ABR-Affinity BioReagents Inc., Golden, CO), diluted 1:500 in 100 μL of PBS-BSA. After this time the cells were washed twice with PBS-BSA and incubated with 100 μL anti-rabbit fluorescein isothiocyanate (FITC)-conjugated antibody (diluted 1:50 in PBS-BSA, Sigma Chemical Co.) for 30 min at 4°C in the dark. The samples were then washed twice in PBS-BSA, fixed with 0.2 mL of 2% w/v paraformaldehyde and re-suspended in 500 μL PBS-BSA. The fluorescence of each sample was recorded using a FACS-Calibur system (Becton Dickinson, Bedford, MA). For each analysis 10,000 events were collected; the green fluorescence of FITC was detected using a 530 nm band pass filter. The percentage of fluorescent cells was calculated by the Cell Quest software (Becton Dickinson). Control experiments included incubation of cells with non-immune isotypic antibodies followed by the appropriate labelled secondary antibodies. Since doxorubicin has a strong autofluorescence which might lead to the misinterpretation of flow cytometry results, we validated FACS experiments by measuring the CRT exposure in biotinylation assays, as reported below.

### Western Blot Analysis

Cells grown at confluence on 60-mm-diameter Petri dishes were washed twice with PBS, then lysed in sample buffer heated at 99°C (25 mmol/L Hepes, 135 mmol/L NaCl, 1% v/v Nonidet P-40, 5 mmol/L EDTA, 1 mmol/L EGTA, 1 mmol/L ZnCl2 and 10% v/v glycerol) and sonicated with one 10 s burst. After centrifugation (13,000 × *g *for 15 min), the protease inhibitor cocktail set III (100 mmol/L AEBSF, 80 μmol/L aprotinin, 5 mmol/L bestatin, 1.5 mmol/L E-64, 2 mmol/L leupeptin, and 1 mmol/L pepstatin; Calbiochem, La Jolla, CA), 2 mmol/L phenylmethylsulfonyl fluoride and 1 mmol/L NaVO_4 _were added to the supernatant. Whole cell extracts containing equal amounts of proteins (30 μg) were separated by SDS-PAGE, transferred to PVDF membrane sheets (Immobilon-P, Millipore, Bedford, MA) and probed with the following antibodies, diluted in 1% w/v PBS-BSA: anti-iNOS (catalogue number 610332 BD Biosciences, blocking peptide: aminoacids 961-1144 of mouse iNOS; rabbit polyclonal, diluted 1:1000), anti-eNOS (catalogue number 610297 BD Biosciences, blocking peptide: aminoacids 1025-1203 of human eNOS; mouse monoclonal, diluted 1:500), anti-nNOS (catalogue number 610308 BD Biosciences, blocking peptide: aminoacids 1095-1289 of human nNOS; mouse monoclonal, diluted 1:500), anti-Pgp (catalogue number sc-8313, Santa Cruz Biotechnology Inc., Santa Cruz, CA; blocking peptide: aminoacids 1040-1280 of human Pgp; rabbit polyclonal, diluted 1:250), anti-MRP3 (catalogue number sc-5774, Santa Cruz Biotechnology Inc.; blocking peptide sc-5774P raised against aminoacids 1-50 of human MRP3; goat polyclonal, diluted 1:250), anti-GAPDH (catalogue number sc-25778, Santa Cruz Biotechnology Inc.; blocking peptide: aminoacids 1-335 of human GAPDH; rabbit polyclonal, diluted 1:500). After an overnight incubation, the membrane was washed with PBS-Tween 0.1% v/v and subjected for 1 h to a horseradish peroxidase-conjugated anti-rabbit (catalogue number 170-6515, Bio-Rad, diluted 1:3000 in PBS-Tween with 5% w/v non-fat dry milk) or anti-mouse antibody (catalogue number 170-6516, Bio-Rad, diluted 1:3000 in PBS-Tween with 5% w/v non-fat dry milk), or to an anti-goat antibody (catalogue number sc-2384, Santa Cruz Biotechnology Inc., diluted 1:1000 in PBS-Tween with non-fat dry milk 5%). The membrane was washed again with PBS-Tween and proteins were detected by enhanced chemiluminescence (PerkinElmer).

To analyse the presence of nitrated proteins, the whole cell extract was subjected to an overnight immunoprecipitation using a rabbit polyclonal anti-nitrotyrosine antibody (catalogue number AB5411, Millipore; diluted 1:100 in blocker non-fat dry milk 1%). Immunoprecipitated proteins were separated by 7% SDS-PAGE, transferred to PVDF membrane sheets and probed with anti-Pgp and anti-MRP3 antibodies, as previously described.

To assess the whole cells content of calreticulin, cells were solubilized in lysis buffer (10 mmol/L Tris-HCl, 100 mmol/L NaCl, 20 mmol/L KH_2_PO_4_, 30 mmol/L EDTA, 1 mmol/L EGTA, 250 mmol/L sucrose; pH 7.5), supplemented with protease inhibitor cocktail set III, 250 mmol/L NaF, 100 mmol/L NaVO_4 _and 100 mmol/L phenylmethylsulfonyl fluoride. The cell lysates were centrifugated at 13,000 × *g *for 5 min at 4°C and the supernatants were centrifuged for 60 min at 100,000 × *g*. The samples were re-suspended in 300 μL lysis buffer of which 15 μL was used to measure the protein content. 10 μg of the cell extracts were separated by 10% SDS-PAGE, transferred to a PVDF membrane sheet and probed with the anti-CRT rabbit polyclonal antibody, diluted 1:1000 in PBS-BSA 1%.

For the detection of calreticulin in the plasma membrane, a cell surface protein isolation kit was used (Thermo Fisher Scientific Inc., Rockford, IL). The cells were washed with PBS, labelled with EZ-Link^® ^Sulfo-NHS-SS-Biotin (Thermo Fisher Scientific Inc.) for 30 min at 4°C, then rinsed with the quenching solution of the kit and washed twice in PBS. The pellet was re-suspended in the lysis buffer provided by the kit, supplemented with protease inhibitor cocktail set III and sonicated on ice with five 1 s bursts. The lysates were incubated 30 min on ice, while vortexing every 5 min for 5 s, then additionally sonicated and centrifugated at 10,000 × *g *for 2 min at 4°C. The isolation of biotinylated cell surface proteins was performed with an affinity chromatography procedure, using the Immobilized neutrAvidin™ Gel (agarose beads; Thermo Fisher Scientific Inc.). The bound proteins were released by incubating the extracts with sample buffer containing 50 mmol/L DTT. Finally the biotinylated proteins were separated by 10% SDS-PAGE, transferred to a PVDF membrane sheet and probed with the anti-CRT rabbit polyclonal antibody as reported.

### Generation of DC from peripheral blood monocytes

Immature dendritic cells were generated as described [32]. Peripheral blood mononuclear cells (PBMC) were isolated from heparinized blood of voluntary healthy donors by standard density gradient centrifugation (Histopaque-1077), washed with RPMI 1640 medium supplemented with 5 mmol/L EDTA and 2% heat-inactivated FBS, and suspended at 5 × 10^6^/ml in RPMI 1640 medium supplemented with 10% FBS. After 2 h incubation at 37°C in a humidified 5% CO_2 _incubator, non-adherent cells were removed, whereas adherent cells (monocytes) were cultured in the above complete medium supplemented with GM-CSF (1000 U/ml) and IL-4 (1000 U/ml). Cytokines were replaced on day 3 and *i*DC were collected on day 6.

### Phagocytosis Assay

The tumor cells were green-stained with PKH2-FITC (Sigma Chemical Co.) according to the manufacturer's instructions. After 4 h the cells were washed and incubated for 20 h at 37°C with 1 × 10^5 ^*i*DC at a 1:1 ratio, and the mixed culture was stained with the phycoerythrin (PE)-conjuganted anti-CD80 antibody (BD Bioscience) for 20 min at 4°C. Phagocytosis of tumour cells by *i*DC was assessed by flow cytometric analysis as the percentage of double-stained (FITC plus PE) versus red (PE) stained cells on a total of 10,000 events, using the DIVA software (FacsCanto system, BD). In parallel a phagocytosis assay was performed by co-incubating *i*DC and tumour cells at 4°C, instead of 37°C. The percentage of double-stained cells obtained after the incubation at 4°C was subtracted from that obtained after a 37°C incubation. The phagocytosis rate was expressed as phagocytic index, calculated as reported by Obeid et al. [4].

### Alloantigen presentation assay

The assay was performed as reported in [38], with minor modifications. DCs that had incubated 20 h with the untreated/treated tumour cells or cultured alone were treated for 2.5 h with 10 μg/ml mitomycin C, estensively washed and then incubated with 1 × 10^5 ^allogenic PBMCs (containing the responder allogenic lymphocytes) at a 15:1 PBMC/DC ratio. Lymphocytes alone and mytomicin-treated tumours (HT29, HT29-dx cells or HT29-iNOS^-^) alone were also plated in parallel sets. Experiments were performed in 96-wells cells culture plates in RPMI-1640 medium supplemented with 10% FBS. To text the extent of DNA synthesis, 1 μCi/ml [methyl-^3^H]-deoxythymidine (PerkinElmer) was added in cells culture at day 4, and after 16 h the samples were harvested onto glass fibre filters. [methyl-^3^H]-deoxythymidine incorporation was evaluated by β-scintillation counting and expressed as count per minute (cpm)/10^5 ^cells. The proliferation index (PI) was calculated as cpm of PBMC incubated with the stimulatory cells/PBMC cultured alone. When applicable, the cpm induced by the tumour alone were subtracted to the cpm obtained with tumour-loaded DC.

### Statistical Analysis

All data in text and figures are provided as mean ± SE. The results were analysed by a one-way analysis of variance (ANOVA) and Tukey's test. *p *< 0.05 was considered significant.

## List of Abbreviations

MDR: multidrug resistance; Pgp: P-glycoprotein; MRPs: multidrug resistance-related proteins; CRT: calreticulin; ER: endoplasmic reticulum; DCs: dendritic cells; NO: nitric oxide; NOS: NO synthase; iNOS: inducible NOS; eNOS: endothelial NOS; nNOS: neuronal NOS; siRNA: small interfering RNA; SNP: sodium nitroprusside; PTIO: 2-phenyl-4,4,5,5,-tetramethylimidazoline-1-oxyl 3-oxide; FBS: foetal bovine serum; PS: penicillin-streptomycin; G6PD: glucose 6-phosphate dehydrogenase; LDH: L-lactate dehydrogenase; BSA: bovine serum albumin.

## Competing interests

The authors declare that they have no competing interests.

## Authors' contributions

SDB and JK carried out the molecular biology experiments, performed the statistical analysis and participated to draft the manuscript; DB isolated the dendritic cells and carried out the phagocytosis assays; EG performed the NOS activity assays; LM and DG participated in the design of the study and in the revision of the manuscript; AB and CR conceived the study, participated in its design and coordination and helped to draft the manuscript. All authors read and approved the final manuscript.
